# Innate immune cell function in statin-treated patients with severe hypercholesterolemia is comparable to normocholesterolemic individuals: A cross-sectional study

**DOI:** 10.1016/j.athplu.2025.11.004

**Published:** 2025-12-04

**Authors:** Harsh Bahrar, Kim Steward, Liesbeth van Emst, Nils Rother, Jeanine E. Roeters van Lennep, Mihai G. Netea, Siroon Bekkering, Niels P. Riksen

**Affiliations:** a Department of Internal Medicine, Radboud University Medical Center, Nijmegen, the Netherlands; b Department of Nephrology, Radboud University Medical Center, Nijmegen, the Netherlands; c Department of Internal Medicine, Erasmus MC Cardiovascular Institute, Erasmus University Medical Center, Rotterdam, the Netherlands; d Department for Immunology & Metabolism, Life and Medical Sciences Institute (LIMES), University of Bonn, 53115, Bonn, Germany

**Keywords:** Hypercholesterolemia, Lipid lowering treatment, Statins, Trained immunity, Inflammation, Innate immune cells, Cytokine production

## Abstract

**Background and aims:**

Hypercholesterolemia is a major risk factor for atherosclerotic cardiovascular disease. Innate immune cells, including monocytes and neutrophils, play important roles in the pathophysiology of atherosclerosis. We previously reported that monocytes from patients with severely elevated low-density lipoprotein (LDL) cholesterol (LDL-c > 4.9 mmol/l) have a hyperresponsive phenotype, which persists even after three months statin treatment. This long-term hyperresponsive innate immune phenotype is termed trained immunity (innate immune memory), and is mediated by persistent enrichment of activating histone modifications leading to higher chromatin accessibility. In this study we investigated the monocyte and neutrophil phenotype of patients with severe hypercholesterolemia treated for 12 months with statins, compared to normocholesterolemic controls.

**Methods:**

In a multicentric cross-sectional study, treatment-naïve patients with severe hypercholesterolemia (defined as LDL-cholesterol >4.9 mmol/L) requiring statin treatment were included. Blood was drawn after 12 months of lipid lowering therapy with statins (n = 15) and compared to healthy normocholesterolemic controls (LDL-c<3.5 mmol/l; n = 18).

We assessed monocyte phenotype with flow cytometry, cytokine production capacity of PBMCs, and H3K4me3 expression on the *TNFA* promotor. In addition, we assessed the neutrophil phenotype and function.

**Results:**

Treatment lowered LDL-c from 5.8 to 2.7 mmol/L. PBMC cytokine production capacity, as well as the expression of H3K4me3 histone mark on the *TNFA* promotor did not differ from the controls (LDL-c 2.6 mmol/L). Although neutrophil CD11b, CD66b, and CD62L expression was the same, production of several granular proteins was lower in patients.

**Conclusions:**

Previous studies reported hyperresponsive monocytes in treatment-naïve patients with LDL-c > 4.9 mmol/l. We now demonstrate that in an independent cohort of patients with LDL-c > 4.9 who are treated for 12 months with lipid-lowering drugs, the monocyte phenotype and function was similar to that of normocholesterolemic controls. In addition, neutrophils phenotype was similar, while the secretion of several granular proteins was lower in the patients.

## Highlights

• Monocytes from hypercholesterolemic patients are known to be hyperresponsive.
• This is associated with enrichment of activating histone modifications.
• We now show normal monocyte responsiveness after one year lipid lowering treatment.
• Also, there were no histone methylation differences compared to control subjects.
• There was no neutrophil activation in treated hypercholesterolemic patients.

## Graphical abstract

**Figure:**
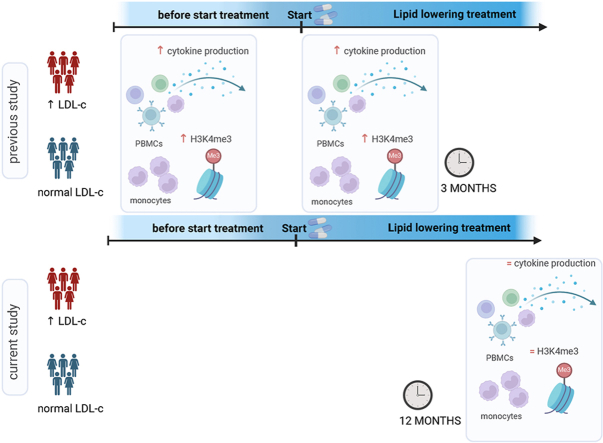

## 1 Introduction

Atherosclerotic cardiovascular diseases (ASCVD) are characterized by a chronic low-grade inflammation of the arterial wall in which innate immune cells such as monocyte-derived macrophages play a central role [1]. Hypercholesterolemia is one of the most important risk factors for ASCVD. Despite lipid lowering treatment, many patients remain at high residual risk to develop ASCVD. Accumulating evidence points to inflammation and immune system activation as important drivers of this residual risk. One specific immune mechanism that contributes to atherosclerosis, is innate immune memory, also called trained immunity [2].

Trained immunity refers to the phenomenon that innate immune cells like monocytes and macrophages, after brief exposure to an inflammatory stimulus, develop a long-term hyperresponsive phenotype. These changes also occur in the bone marrow myeloid progenitor cells, which explains the durable effects of trained immunity in vivo [3]. In vitro, trained immunity can be induced by endogenous atherogenic molecules such as oxidized low density lipoprotein (LDL) [4], and lipoprotein (a) [5]. Recent experimental studies in murine models of atherosclerosis unequivocally showed that intermittent high fat diet also induces trained immunity, which subsequently accelerates atherosclerosis by lasting changes in macrophage [6], and neutrophil function [7]. In addition to monocytes and macrophages, also other innate immune cells contribute to atherogenesis, including neutrophils [8]. Interestingly, neutrophils can also mount a trained immunity response [9], and persistent neutrophil activation is critical for the pro-atherogenic effects of high fat diet-induced trained immunity [7].

Trained immunity is mediated by metabolic reprogramming and epigenetic modifications such as enrichment of the activating histone modification H3K4me3, which tags inflammatory genes for enhanced and more rapid expression after stimulation [10]. Interestingly, the mevalonate pathway, which is critical for cholesterol biosynthesis, also contributes to this process, and in vitro studies have shown that trained immunity can be prevented by statins [11]. Statin treatment, however, cannot revert the hyperresponsive trained phenotype once this has established. This is illustrated by our previous study in which we demonstrated the presence of augmented PBMC cytokine production capacity and enrichment of H3K4me3 on the *TNFA* gene promoter, as markers for trained immunity, in patients with severe hypercholesterolemia (defined as LDL-c > 4.9 mmol/l), which persisted despite three months lipid-lowering therapy with statins [12]. There are no previous data on neutrophil function and phenotype in patients with hypercholesterolemia.

In the current study, we aimed to investigate whether the hyperresponsive trained immune phenotype in monocytes from patients with severe hypercholesterolemia persisted after 12 months of statin treatment compared to healthy controls. This 12-month timepoint is based on previous studies showing that monocyte trained immunity induced by Bacillus Calmette-Guérin vaccination was present three months after vaccination, but after 12 months this pro-inflammatory phenotype was almost completely reversed [13]. Therefore, we hypothesized that 12 months of lipid lowering is sufficient to normalize monocyte function. To test this hypothesis, we performed extensive immunophenotyping of monocytes from patients with LDL-c concentrations >4.9 mmol/l after 12 months lipid lowering with statins and compared this to healthy normocholesterolemic subjects. In addition, we also compared the neutrophil function of patients and controls.

## 2 Materials and methods

### 2.1 Study design

This study is an observational multicentre study, including patients with severe hypercholesterolemia (defined as untreated LDL-cof >4.9 mmol/l, who require lipid lowering treatment according to the treating physician. We planned to investigate the effect of long term (12 months) statin treatment on the innate immune phenotype and compare it with healthy controls with normal LDL-c values. Importantly, there is no overlap in patients or controls between the current study and our previous study in which we used similar in- and exclusion criteria [12].

We designed a prospective case-control study with repeated immunological assessments after 3 months and 12 months of treatment. However, our study started in the middle of the COVID19 pandemic during a national lockdown, hence it was not possible to include the healthy controls in the same time period as the patients. Therefore, we include the healthy controls in the same period as the 12-months time point of the patients (Supplementary Fig. 1). Unfortunately, it was recently convincingly shown that both lock-downs and COVID-vaccinations have a strong effect on PBMC cytokine production capacity, which is the primary parameter of the current study [14]. Because these confounders hinder the longitudinal comparison in the patient group, we switched to a cross-sectional design in which we only compared the patients with hypercholesterolemia at the 12-months treatment time point to the normocholesterolemic control subjects, to allow for comparison of samples in the same recruitment period and exclude these COVID-related confounding effects.

This study was approved by the Medical Ethics Committee of the Radboud University Medical Centre, Nijmegen and Erasmus Medical Centre, Rotterdam, The Netherlands (NL72155.091.20). The study was conducted in compliance with the principles of the Declaration of Helsinki.

### 2.2 Study population

The patients and healthy controls were recruited at Radboud university medical centre (RUMC) Nijmegen, and Erasmus Medical Centre (EMC) Rotterdam, The Netherlands.

### 2.3 Inclusion and exclusion criteria

All participants were 18 years or older. Patients were eligible to participate if their LDL-c levels were above 4.9 mmol/l, they did not receive cholesterol lowering treatment for the last 12 months and they had no previous cardiovascular event. We selected healthy controls with LDL-c below 3.5 mmol/l, with no prior cardiovascular events and no cholesterol lowering drugs.

Participants were not eligible if they were unable to provide written informed consent, had a current treatment for malignancy, had an acute or chronic infection with fever at the time of participation, had clinically significant infections within 1 months prior to study entry (defined as fever >38.5 °C), had vaccination within 1 months prior to study entry, had a medical history of any disease associated with immune deficiency (either congenital or acquired, including chemotherapy, chronic steroid use, organ transplantation), used chronic anti-inflammatory drugs such as NSAIDs (acetylsalicylic acid <100 mg/day excluded).

For the patients during the follow up at the indicated time points, we made sure that there were at least 4 weeks in between blood drawing and events like recent vaccinations and infections.

### 2.4 Blood sampling and lipid measurement

At each timepoint venous blood was collected into BD Vacutainer® K2EDTA (50 ml) and BD Vacutainer® serum separator tube (5 ml). To ensure that there was no difference in the isolation of immune cells, all the samples (collected at Radboudumc and ErasmusMC) were drawn in the morning and the isolation started 4 h after the blood draw, in this way the samples could be transported during the morning to Radboudumc for the isolation.

Two EDTA vacutainers (20 ml) and the serum separator vacutainer were centrifuged for 10 min at 3800 rpm at room temperature to collect the plasma and sterile serum, which was stored at −80 °C. Whole blood composition was measured with the Sysmex-XN 450 hematology analyzer. Total cholesterol, triglycerides (TG), and high-density lipoprotein (HDL) cholesterol levels were analyzed with commercially available enzymatic methods, and LDL-c was calculated with the Friedewald equation [15].

### 2.5 Flow cytometry

Flow cytometric analyses were performed on whole blood with panels for monocytes and neutrophils.

EDTA (600μL) whole blood was taken, and the red blood cells were lysed in the dark with the BD Pharm Lyse buffer (15 min) at room temperature. Cells were washed with FACS buffer (1 % BSA in PBS with 2 mM EDTA), resuspended in FACS buffer with Human TruStain FcX (Biolegend, San Diego, CA, USA) Fc block. The specific antibodies used to characterize monocytes and neutrophils are shown in Supplementary Table 1. Supplementary Fig. 2 shows the gating strategy for monocytes and neutrophils.

### 2.6 Cell isolation

#### 2.6.1 Peripheral blood mononuclear cell (PBMC)

Blood was diluted in PBS (phosphate buffered saline, Gibco) and peripheral blood mononuclear cells (PBMCs) were isolated using Ficoll‐Paque PLUS (Cytiva) density gradient centrifugation for 30 min at 615g (no brake or acceleration, RT). The PBMCs with platelet rich plasma (PRP) were collected and washed with PBS containing 0.1 % human pooled serum and 1 mM UltraPure EDTA (0.5 M, pH 8, Life Technologies) at 190g for 15min, RT, to separate the PRP from the PBMCs. The PBMCs were then washed two times with cold PBS and resuspended in RPMI 1640 Dutch-modified culture medium supplemented with 1 mM pyruvate (Invitrogen), 2 mM glutamine (Invitrogen), 50 μg/mL gentamicin (Centrafarm). The PBMC composition was assessed at the Sysmex-XN450 hematology analyzer.

#### 2.6.2 Polymorphonuclear neutrophil (PMN) isolation

The pelleted cells after PBMC Ficoll separation (containing neutrophils and red blood cells) were resuspended in 50 ml hypotonic lysis buffer (155 mM NH4Cl, 10 mM KHCO3) twice for 15 and 10 min on ice to lyse red blood cells and centrifuged after each incubation. Afterwards, the PMNs were washed twice in PBS and resuspended in RPMI 1640 medium without phenol red (Gibco, 32404014) supplemented with 50 μg/mL gentamicin (Centrafarm), 2 mM glutamax (Gibco), and 1 mM pyruvate (Gibco). Neutrophil cell number and purity were assessed with Sysmex-XN450 Hematoanalyzer.

#### 2.6.3 Platelet rich plasma (PRP) collection and isolation of platelets

The PRP was centrifuged at 2500g, 5 min, 4 °C and very gently resuspended in previously described RPMI 1640 Dutch-modified culture medium without phenol red. Platelet number and purity were assessed by the Sysmex–XN450 hematology analyzer. Platelets were used for the NOX-independent NET-assays (see below).

#### 2.6.4 Monocyte isolation

Monocytes were isolated from the PBMCs using the MACS pan-monocyte isolation kit (Miltenyi Biotec) according to manufacturer's protocol. The cells were resuspended in previously described supplemented RPMI 1640 Dutch-modified culture medium and assessed for number and purity by the Sysmex- XN 450 hematology analyzer.

### 2.7 Stimulation assays

#### 2.7.1 PBMC stimulation

The isolated PBMCs were stimulated (500.000 cells per well) for 24 h in duplicate in round‐bottom 96‐well plates (Corning, NY, USA) with RPMI (negative control), 10 ng/mL lipopolysaccharide (LPS; Sigma-Aldrich, St. Louis, MO, USA) from *Escherichia coli*, purified as previously described [16], 10 μg/mL Pam3Cys (P3C; EMC Microcollections, Germany), 1x106/mL heat killed *Candida albicans* conidia (*C. albicans*; UC 820, in house), 100 μg/mL polyinosinic:polycytidylic acid (PolyI:C; Invivogen, San Diega, CA, USA), 300 μg/mL monosodium urate crystals (MSU; Sigma-Aldrich), and MSU + LPS (MSU prepared as described before [17]). After incubation at 37 °C with 5 % CO_2_ for 24 h, the plates were centrifuged at 348 g, the supernatants collected, and stored at −80 °C until cytokine measurements.

#### 2.7.2 Neutrophil stimulation

The isolated neutrophils were stimulated (500.000 cells per well) in duplicate in flat bottom 96-well plates (Corning, NY, USA) for 4 h with culture medium only as negative controls, 1 μg/ml LPS, 10 μg/ml P3C, 1 μM Nigericin, 50 nM phorbol-12-myristate-13-acetate (PMA), 300 μg/mL MSU, and 300 μg/mL MSU combined with 1 ng/mL LPS for 4 h at 37 °C, 5 % CO_2_. . After incubation, the plate was centrifuged at 348 g for 8 min at RT, and 180 μl of cell culture supernatant was collected, and stored at −80 °C.

### 2.8 Neutrophil extracellular traps (NET) assays

#### 2.8.1 NOX-dependent NET assay

Neutrophils were plated (200.000 cells per well) on flat-bottom 96-well plates (Corning, NY, USA) at 37 °C with 5 % CO_2_ for 20 min to attach. Afterwards, the medium was removed and the attached cells were stimulated in quadruplicate with culture medium only (as control), Nigericin (1 μM), Ethanol (control for nigericin), PMA (50 μM) for 3 h at 37 °C, 5 % CO_2_.

#### 2.8.2 NOX-independent NET assay

The isolated platelets (1x10^8^) were stimulated either with 156uM Thrombin Receptor Activator Peptide 6 (TRAP6, Sigma) or left unstimulated for 30 min at 37 °C, 5 % CO_2_ in round bottom non-stick tubes (corning 352063, Fisher Scientific). Neutrophils were plated (200.000 cells per well) on flat-bottom 96-well plates (Corning, NY, USA) at 37 °C with 5 % CO_2_ for 20 min to attach while the platelets were incubating.

The neutrophils were then stimulated in quadruplicate with culture medium only (as control), unstimulated platelets, and platelets stimulated with TRAP6 for 1 h at 37 °C, 5 % CO_2_.

After the incubation in both NOX dependent and independent assay, the neutrophils were washed gently twice with 37 °C PBS, and culture medium supplemented with 5 U/ml micrococcal nuclease (MNase, Worthington biochemical corporation) was added for 20 min at 37 °C, 5 % CO2 to detach/digest the NETs. MNase was then inactivated by vortexing, and the supernatant containing partially digested NETs were collected after centrifugation and stored at −80 °C until future measurement.

#### 2.8.3 Measurement of DNA concentration in NETs with Sytox Orange

Sytox Orange Nucleic Acid Stain (Life Technologies) was used to quantify the DNA concentration of NOX-dependent and NOX-independent NETs via fluorescence measurement with excitation and emission of 530/560 nm using the BioTek Synergy HT multi-reader. All the measurements were done in duplicate.

### 2.9 Neutrophil reactive oxygen species (ROS) assay

The neutrophil ROS was measured based on luminol (5-amino-2,3, dihydro-1,4-phtalazinedione)-enhanced luminescence assay. Neutrophils (200.000 cells per well) were added on an white flat-bottom 96-well plates (Corning, NY, USA) and stimulated in quadruplicate with culture medium only (as control), serum-opsonized zymosan and PMA. Once upon adding the luminol, the chemiluminescence was measured for 1 h at 37 °C in a BioTek Synergy HTreader. The integral of relative luminescence units per second (RLU/sec) was measured.

### 2.10 Circulating plasma protein and cytokine measurements

Circulating plasma protein concentration (hCRP, S100A8/9, ELA2, NGAL, MPO) or cytokines upon stimulation (IL-1Ra, IL-1β, IL-6, IL-8, IL-10, TNF, S100A8/9, NGAL, MPO) were measured using DuoSet ELISA kits (Bio-Techne/R&D Systems) following the manufacturer's instructions. For identifiers of ELISA kit (Supplementary Table 2).

### 2.11 Chromatin immunoprecipitation (ChIP) and qPCR

For the assessment of H3K4me3 (activating histone mark) at least 2x10^6 freshly isolated purified monocytes were fixed with methanol-free 1 % formaldehyde for 8min followed by 1.25M Glycin for 3min and then centrifuged and snap frozen in pellet and stored at −80 °C until future measurement.

The cell pellets were equalized per cell number (2x10^6 per sonication cycle) using the ultra-sonicator S220 (Covaris), and the sonicated material was aliquoted and snap frozen for ChIP at a later timepoint. A small portion of every sample was checked for the purity at base pair size on an agarose gel.

The chromatin was divided into ChIP and input samples. Dilution buffer (1.0 % Triton, 1.2 mM EDTA, 16.7 mM Tris (pH 8.0), 126 mM NaCl, 1x protease inhibitor in MilliQ) was added to the ChIP samples, along with 1ug of ChIP grade H3K4me3 antibody (9751S, Cell Signaling Technology) and rotated overnight at 4 °C. Magnetic protein A/G beads (Dynabeads) were used for capture. The beads were washed twice in dilution buffer containing 0.15 % SDS and 0.1 % BSA. The washed beads were added to ChIP samples and rotated for 1 h at 4 °C. The bonded bead chromatin suspension was washed one time with washing buffer 1 (2 mM EDTA, 20 mM TRIS (pH 8.0), 1 % Triton, 0.1 % SDS, 150 mM NaCl in MilliQ), twice with washing buffer 2 (similar as washing buffer 1, but with 500 mM NaCl) and twice with washing buffer 3 (1 mM EDTA, 10 mM Tris (pH 8.0) in MilliQ).

The chromatin was eluted from the beads in elution buffer (1 % SDS, 0.1M NaHCO3 in MilliQ) and rotated for 20 min at RT. Input samples were diluted 12 times in elution buffer. After adding proteinase K (0.1 mg/ml) and NaCl (0.2 M) to all samples, they were de-crosslinked for at least 4 h on a shaking heat block (at 65 °C, 1000 rpm). MinElute PCR purification columns (Qiagen) were used to purify DNA fragments. DNA fragments were stored at 4 °C until analysis by qPCR.

SYBR green method was used for the qPCR with the primers covering the whole *TNFA* promotor region mentioned in the Supplementary Table 3. Samples were analyzed by a comparative Ct method in which ChIP was compared against input samples. Myoglobin was used as a negative locus control and GAPDH as a positive locus control for H3K4me3.

### 2.12 Statistics

Unless otherwise indicated all data are presented as mean ± SD. We compared all immunological parameters between treated patients and normocholesterolemic controls using independent sample T-tests, or Mann-Whitney U tests, where appropriate. *P* below <0.05 was regarded as statistically significant. All the statistical testing was performed with IBM SPSS statistics version 30 and GraphPad Prism version 10. Sample size was based on our previous study in a comparable patient population [12].

## 3 Results

### 3.1 Baseline characteristics

**Fig. 1:**
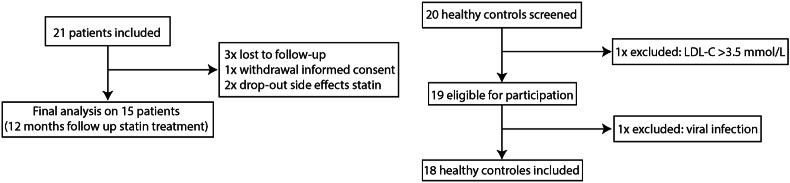
Consort diagram, showing the selection of subjects and exclusion reasons.

We included twenty-one patients in the study. Six patients dropped out, because they were lost to follow-up (n = 3), due to withdrawal of informed consent after vasovagal reaction on blood drawing (n = 1), and due to the side effects of statins (n = 2). In total, fifteen patients completed the 12 month follow up after start of statin treatment. A total of twenty healthy controls were screened, from which nineteen were eligible to participate in the study, and one dropped out due to viral respiratory tract infection after the screening (Fig. 1). The baseline characteristics and plasma lipid levels, both before treatment as well as after 12-month statin treatment, are shown in Table 1.

**Table 1:** **Baseline characteristics of the participants.** Data is presented as mean ± SD. *p*-values represent comparison with healthy control patients; *p* < 0.05 was considered significant, using the unpaired T-test statistical test. Please note that weight, BMI, blood pressure and heart rate were not assessed again after 12 months treatment.

	Healthy controls (n = 18)	Patients untreated (n = 15)	*p*-value	Patients treated (n = 15)	*p*-value
	Mean ± SD	Mean ± SD		Mean ± SD	
**Age (yr)**	44 ± 9	43 ± 12	0.79		
**Sex, n (% female)**	13 (72 %)	11 (73 %)	0.95		
**Height (cm)**	177 ± 12	170 ± 6	0.04		
**Weight (kg)**	74 ± 12	79 ± 15	0.29		
**BMI (kg/m2)**	23.9 ± 3	27.5 ± 4.7	0.03		
**Waist (cm)**	84 ± 10	96 ± 16	0.05		
**Systolic blood pressure (mmHg)**	120 ± 9	129 ± 12	0.05		
**Diastolic blood pressure (mmHg)**	74 ± 7	81 ± 11	0.08		
**Heart rate (beats per min)**	66 ± 12	82 ± 16	0.01		
**Total Cholesterol (mmol/L)**	4.7 ± 0.6	7.6 ± 1.1	<0.0001	4.5 ± 1.1	0.6
**LDL-Cholesterol (mmol/L)**	2.6 ± 0.5	5.8 ± 0.9	<0.0001	2.7 ± 1.0	0.8
**Triglycerides (mmol/L)**	0.9 ± 0.3	1.5 ± 0.8	0.004	1.1 ± 0.5	0.09
**HDL-Cholesterol (mmol/L)**	1.7 ± 0.4	1.4 ± 0.3	0.06	1.4 ± 0.3	0.02
**hsCRP (mg/l)**	0.6 ± 0.5	1.1 ± 0.9	0.13	1.3 ± 1.3	0.16

### 3.2 Lipid-lowering treatment

The choice of the statins was at the discretion of the treating physician. The prescribed statins are summarized in Supplementary Table 4. Most prescribed statin at 12 months was rosuvastatin (n = 10/15), six patients received ezetimibe in combination with statin therapy. At 12 months two patients had discontinued statin therapy because of side effects.

### 3.3 Circulating white blood cells and high-sensitivity C-reactive protein (hsCRP)

When comparing the treated patients with the normocholesterolemic controls, there was no significant difference in circulating leukocyte count (6.1 ± 1.7 versus 5.7 ± 1.6 ∗10^9^/l, P = 0.57), leukocyte differentiation (neutrophil count 3.5 ± 1.3 versus 3.2 ± 1.2, P = 0.45; lymphocytes 2.0 ± 0.6 versus 1.8 ± 0.6, P = 0.23, monocytes 0.4 ± 0.1 versus 0.5 ± 0.2, P = 0.41), and hsCRP (1.3 ± 1.3 versus 0.6 ± 0.5, P = 0.16). At baseline, before initiation of statins in the patients, the circulating leukocyte numbers was 6.0 ± 1.6 (neutrophils 3.5 ± 1.2; lymphocytes 1.9 ± 0.6; monocytes 0.4 ± 0.1).

### 3.4 No differences in monocyte phenotype and function after 12 months statin treatment compared to normocholesterolemic controls

#### 3.4.1 Monocyte subsets and surface activation markers

**Fig. 2:**
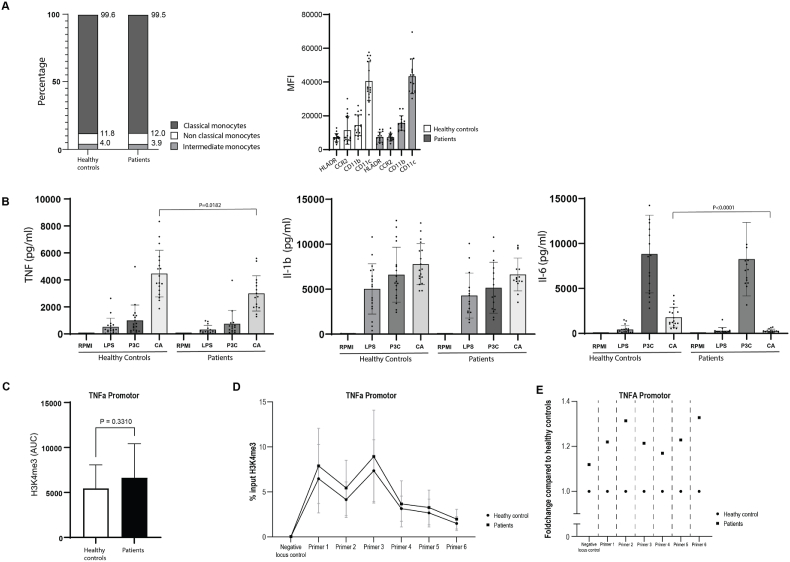
**PBMCs cytokine production capacity and monocyte phenotype** (A) Monocytes subset distribution and surface marker expression (in MFI) in patients and healthy controls. (B) PBMC cytokine production capacity. (C) Area under the curve (AUC) of histone trimethylation at H3K4 site of TNF promotor region. Data are presented as mean ± SD. *p*-value <0.05 was considered significant, using the Mann-Whitney U statistical test. (D) The percentage input of H3K4me3 on the *TNFA* promotor region (shown are negative control locus and 6 different primers) in patients compared to healthy controls. (E) Fold change percentage of input relative to healthy controls.

Flow cytometry did not reveal significant difference in monocyte subsets, nor in the MFI of HLA-DR, CCR2, CD11b, CD11c on positive cells (Fig. 2a). There were also no differences in the percentages of CCR2+ (89 ± 4 % versus 89 ± 4 for patients versus controls, P = 0.61), CD11b+ (97 ± 2 versus 96 ± 3, P = 0.94) and CD11c+ (100 ± 0 versus 100 ± 1, P = 0.11) monocytes.

#### 3.4.2 Cytokine production capacity

We have previously described that LPS-induced TNF, IL-6, and IL-1b production, P3C-induced IL-6 production, and *Candida albicans*-induced TNF and IL-6 production were higher in treatment naïve patients with severe hypercholesterolemia [12]. Fig. 2b shows the cytokine production capacity of PBMCs after 24 h stimulation in patients after 12-month statin treatment and healthy controls. At this time point there were no significant differences in TNF, IL6 and IL1b production upon stimulation with LPS and P3C. Stimulation with C. Albicans resulted in significantly lower TNF and IL-6 production in patients, compared to controls (*p* = 0.0182 and *p* < 0.0001, respectively). In addition, cytokine production in response to MSU, LPS + MSU, and Poly I:C was not different between the groups (Supplementary Table 5).

#### 3.4.3 Histone methylation marks

We previous reported that the functional hyperresponsiveness of monocytes from untreated patients with severe hypercholesterolemia was associated with enrichment of the activating histone modification H3K4me3 on the *TNFA* gene promoter [12], a typical biomarker associated with trained immunity. Therefore, we now performed chromatin immunoprecipitation with qPCR on isolated monocytes from patients and healthy controls to assess H3K4me3 on the *TNFA* promotor region. After twelve months of lipid-lowering with statins, the treated patients had similar levels of H3K4me3 on the *TNFA* promoter region compared to healthy controls (Fig. 2c), which is in line with the cytokine production capacity that is similar between groups. In Fig. 2d we show the percentage H3K4me3 on the six different primer loci together with a negative locus control on the *TNFA* promoter region. The raw percentage of input data are given in Supplementary Table 6. There were no significant differences between the two groups, which is in line with the cytokine production capacity. In Fig. 2e we show the fold change of the percentage input relative to healthy controls for each primer and the negative locus control.

### 3.5 Effect of statin treatment on neutrophil granular protein release

#### 3.5.1 Neutrophil activation markers

**Fig. 3:**
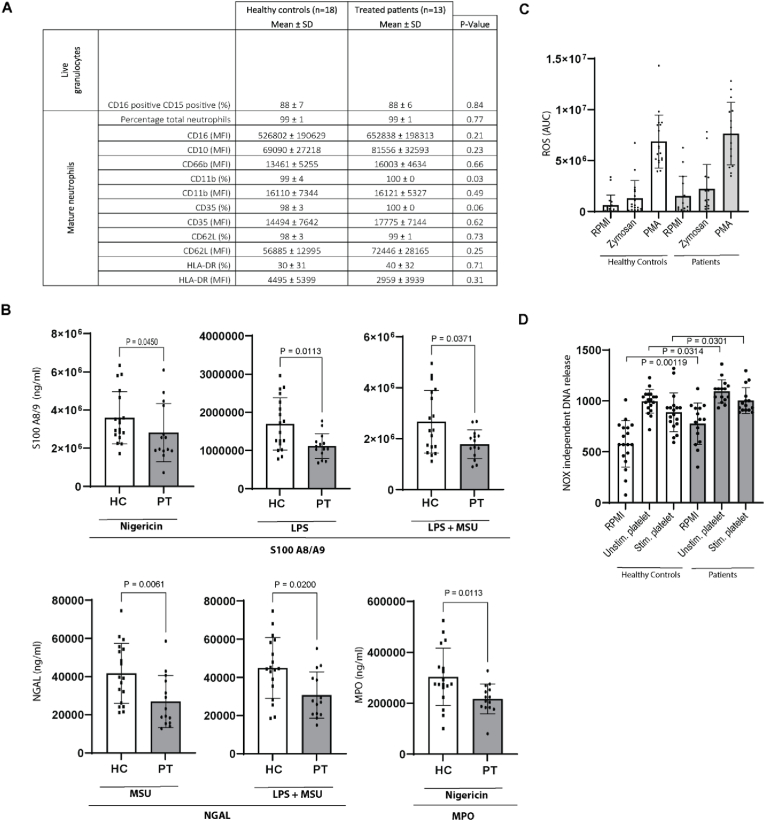
**Neutrophil phenotype and function.** (A) Neutrophils surface marker expression in percentage of positive cells and as MFI value. (B) Neutrophil secretion of MPO, S100A8/A9, NGAL upon stimulation with Nigericin, MSU, LPS and LPS + MSU. (C) ROS production capacity of neutrophils upon stimulation with RPMI, Zymosan and PMA (D) DNA released via the NOX independent NETs formation. Data are presented as mean ± SD. *p*-value <0.05 was considered significant, using the Mann-Whitney U statistical test.

Since 99 % of the neutrophils were mature neutrophils in treated patients and controls, we only focused on this subset. After twelve months statin treatment, patients with hypercholesterolemia have similar expression of cell surface activation markers compared to healthy controls, both for the percentage of positive cells, as well for the MFI, for the markers CD16, CD10, CD66b, CD11b, CD35, CD62L, and HLA-DR. (Fig. 3a).

#### 3.5.2 Neutrophil granular protein release and cytokine production

Neutrophils have four different types of granules containing specific molecules such as myeloperoxidase (MPO), neutrophil gelatinase-associated lipocalin (NGAL), neutrophil elastase (NE) and S100A8/9 (calprotectin). The content of the granules is released [18] depending on PRR (pattern recognition receptor) that is stimulated. To assess this granule release, we isolated neutrophils and stimulated them ex vivo with PRR ligands for 4 h. Interestingly, in comparison with healthy volunteers, neutrophils of treated patients secreted significantly less S100 A8/A9 and NGAL upon stimulation with LPS, LPS + MSU and nigericin, an NLRP3 inflammasome inducer (Fig. 3b). It is important to mention that for the nigericin-stimulated NGAL secretion, that the negative control group with ethanol stimulation also secreted less NGAL, so probably this is not specific for nigericin. In the nigericin stimulated neutrophils we also measured less MPO secretion (Fig. 3b). There was no difference in LPS or PMA stimulated neutrophils and IL-8 production between the two groups (Supplementary Table 7).

There were no significant differences in circulating plasma granule proteins between treated patients and healthy controls (ELA2 23142 ± 5428 ng/ml versus 22627 ± 5688, P = 0.79; MPO 23373 ± 5880 versus 21446 ± 5481, P = 0.34; S100 278683 ± 168243 versus 225073 ± 121765, P = 0.51; NGAL 74836 ± 19625 versus 76182 ± 16562, P = 0.83).

#### 3.5.3 Reactive oxygen species (ROS) capacity and neutrophil extracellular trap (NET) formation

Two major mechanisms by which neutrophils can fuel inflammation are ROS production and NET formation. To assess the ROS production capacity of neutrophils, we measured the ROS production upon stimulation with RPMI (as control medium), Zymosan and PMA. Statin-treated patients with hypercholesterolemia showed no significant differences in ROS production capacity compared to healthy controls (Fig. 3c).

NET formation is a form of programmed cell death in which neutrophils release their intracellular components as a DNA web that induces inflammation. There are two forms of NET formation [19], a NADPH oxidase (NOX) dependent- and independent NET formation (induced by activated platelets). To quantify this, we induced ex vivo NOX dependent and NOX independent NETs and measure the DNA that was released.

The NOX dependent NETs were similar between the two groups upon stimulation with Nigericin (1380 ± 199 pg/ml in the treated patients versus 1321 ± 287 in controls, P = 0.70) and PMA (951 ± 222 pg/ml versus 956 ± 215, P = 0.89). This is in line with the ROS production capacity, which is also NADPH dependent. In the NOX independent NETs however treated patients had significantly more DNA release in every condition compared to healthy controls (Fig. 3d).

## 4 Discussion

Monocytes and their bone marrow progenitors can develop a long-term pro-inflammatory phenotype after exposure to endogenous atherogenic stimuli such as oxLDL [2,4]. This hyper-responsive phenotype is called trained immunity, and this mechanism of hypercholesterolemia-induced trained immunity augments atherosclerosis development in experimental models [7]. We previously showed that monocytes from treatment-naive patients with severe hypercholesterolemia (defined as LDL-c concentrations >4.9 mmol/l) have an exaggerated PBMC cytokine production capacity, associated with an enrichment of the activating histone modification H3K4me3 [12]. Importantly, this persisted for three months after normalization of cholesterol levels with statins, indicating a trained immunity phenotype [12]. In the current study, we showed in an independent cohort of patients with LDL-c concentrations >4.9 mmol/l that this monocyte hyperresponsiveness is not present anymore after twelve months of lipid lowering therapy, compared to a healthy normocholesterolemic control group. Correspondingly, H3K4me3 levels at the *TNFA* gene promotor were similar. We expanded the functional analyses also to neutrophils, another innate immune cell type recently suggested to be involved in the atherosclerosis process [8]. Similar to monocytes, neutrophils did not show a hyperinflammatory phenotype in these statin-treated patients, but rather a lower neutrophil release of various granular proteins.

The main focus of our study was the investigation of the trained immunity phenotype in monocytes of patients with hypercholesterolemia treated with lipid-lowering drugs. Since various recent experimental studies unequivocally showed that trained immunity, induced by either alternating high fat-diet [20], hyperglycemia [21], or by an acute myocardial infarction [22] accelerates atherosclerosis development, it is very important to be informed about the duration of the trained immune monocyte phenotype in patients with hypercholesterolemia following initiation of treatment.

What do we know about the duration of trained immunity in humans in vivo? These data are only available for trained immunity induced by BCG vaccination. Healthy individuals receiving the BCG vaccine showed a trained immune monocyte phenotype at 2 weeks and 3 months after vaccination. The long duration is explained by the fact that trained immunity occurs in the bone marrow hematopoietic stem and progenitor cells. This was recently investigated after in humans three months after BCG vaccination, revealing persistent changes in gene expression primarily within uncommitted stem cells, and changes in chromatin accessibility being more prevalent in differentiated progenitor cells such as common myeloid progenitors and granulocyte-monocyte progenitors [23]. However, at 12 month after the vaccination, the functional hyperresponsiveness was almost completely reversed [13]. Therefore, we hypothesized that after twelve months of lipid lowering, the hyperresponsive monocytes phenotype returns to the level of normocholesterolemic control subjects. Indeed, we observed similar cytokine production capacity profiles, which was associated with similar presence of H3K4me3 on the *TNFA* gene promotor, in patients treated for 12 months with statins. These observations are in line with clinical observations that the cardiovascular risk of patients with familial hypercholesterolemia is completely lowered to that of the general population by statin therapy [24]. Interestingly, TNF and IL-6 production after candida albicans stimulation was lower in the treated patients than in the controls (Fig. 2B). This suggests a potential direct effect on dectin-1, the receptor that binds β-glucans, but this is not known.

It's important to realize that our findings do not exclude that there are persisting differences in systemic inflammation, which is largely unrelated to monocyte function [25]. We only measured hsCRP, which was not different between the treated patients and controls, but we did not include more markers of systemic inflammation.

While our previous study in patients with severe hypercholesterolemia was restricted to monocyte function, we now also expanded our immune assays to neutrophil function. Accumulating evidence shows that neutrophils are causally involved in the pathophysiology of atherosclerosis [8,26]. In addition, neutrophils can also mount a hyperresponsive trained immune phenotype by epigenetic reprogramming of their bone marrow progenitors [9]. Moreover, we recently showed that the acceleration of atherosclerosis in a mouse model of intermittent high fat-induced trained immunity was dependent on neutrophil activation [7].

Data on the relation between LDL-c and neutrophil function are sparse [27]. In mice, a high fat diet increases bone marrow neutrophil mobilization, due to increased CXCL-1 and -2 expression [28]. In the UK Biobank, there was no significant association between LDL-c and neutrophil counts [29]. To the best of our knowledge, there is no data on neutrophil function in patients with severe hypercholesterolemia. *In vitro*, ox-LDL activates migration and degranulation of isolated human neutrophils [30] and increases NET formation [31].

Cholesterol crystals can induce NETs formation in human neutrophils [32] and a recent study [33] showed that in hypercholesterolemic mice there was an impaired clearance of NETs, which resulted in a prolonged inflammation. In our study we did not see any difference in NOX-dependent NETs between treated patients and healthy controls. Interestingly, NOX-independent NETs were higher in treated patients, even in the unstimulated condition. This could be due to a lasting effect of previous hypercholesterolemia, or could be due to a direct effect of statins, although we could not find any previous evidence for this. Neutrophils of treated patients secreted less MPO upon nigericin and less S100 A8/A9 (calprotectin) and NGAL upon stimulation with LPS, LPS + MSU, nigericin compared to the healthy controls. A possible mechanism could be related to pleiotropic effects of statins independent of LDL-c levels. Statins inhibit the mevalonate pathway leading to a decreased production of geranylgeranyl pyrophosphate (GGPP) and its binding to Rho-GTPases (Rac2) [34,35]. A recent study showed that Bone marrow neutrophils from Rac2^−/−^ mice showed less primary granule (such as MPO) exocytosis, but not secondary or tertiary granule exocytosis compared to wildtype mice [36].

In conclusion, the main finding of this study is that monocyte phenotype and cytokine production capacity is similar in 12 month statin-treated patients with severe hypercholesterolemia compared to normocholesterolemic individuals. This finding is of great importance in light of our previous finding that monocyte function is enhanced in treatment-naïve patients with severe hypercholesterolemia and does not return to baseline by three months of statin treatment, because of persistent enrichment of the H3K4me3 on the cytokine gene promoters [12]. Now, after twelve months treatment, there was no difference anymore in H3K4me3 levels compared to normocholesterolemic individuals.

New compared to our previous study, is that we also expanded our analyses to neutrophils. In the statin-treated patients neutrophils do not have a more pro-inflammatory phenotype compared to normocholesterolemic individuals.

### 4.1 Limitations

This study has several limitations. First, although initially designed as prospective study in which we compared immune function before and after 12 months of lipid lowering, it was only possible to validly compare normocholesterolemic control individuals to the patients after 12 month of lipid lowering. This was due to the fact that blood draws for these groups were performed in the same time window, whereas the baseline blood draw of the patients with hypercholesterolemia was in an earlier time window, starting during the first Dutch national lock-down in the COVID19 pandemic. This is cumbersome, as a recent study from our lab, that was conducted at the same time in the Netherlands [14], showed unequivocally that lockdowns, as well as vaccinations, profoundly influenced PBMC cytokine production capacity, the main immunological read-out of trained immunity. These limitations do not undermine the validity of our main finding that in patients with hypercholesterolemia who are treated with lipid-lowering drugs for twelve months, the monocyte phenotype and function is comparable to that of normocholesterolemic healthy controls, while neutrophil granule protein production is even less. Another limitation is that the inclusion criterium of our study was an LDL-c concentration of >4.9 mmol/l, but we did not take into account any potential (genetic) diagnosis of familial hypercholesterolemia. Per definition, all individuals with LDL-c > 4.9 mmol/l have a Dutch Lipid Clinical Network score of 3 (“possible FH”) or higher. We did not perform genetic testing for FH in all participants. In mice, trained immunity occurs already after 4 weeks of a high-fat/high-cholesterol diet [20]. Therefore, for this particular research questions, the distinction between hypercholesterolemia due to FH (in which case hypercholesterolemia is present from early age on) or due to polygenetic causes is not essential. However, it is important to realize that our results cannot be extrapolated to patients with FH. A third limitation is the limited sample size. Although this sample size is usually sufficient to demonstrate significant differences in cytokine production capacity between two different groups [37,38] it is important to validate our results in independent patient groups. Finally, we only assessed one particular epigenetic marker that is characteristic of trained immunity (H3K4me3), because we previously reported that this specific marker was higher in patients with hypercholesterolemia [12]. For future studies, it would be interesting to also include other epigenetic markers, as well as the activity of metabolic pathways, which are also different in trained immunity.

## Author contributions

**Harsh Bahrar:** Conceptualization, Methodology, Formal analysis, Investigation, Resources, Data Curation, Writing – original draft, Visualization, Project administration. **Kim Steward:** Investigation, Resources, Project administration. **Liesbeth van Emst:** Resources, Investigation. **Nils Rother:** Writing – review & editing. **Jeanine E. Roeters van Lennep:** Conceptualization, Resources, Writing – review & editing. **Mihai G. Netea:** Writing – review & editing. **Siroon Bekkering:** Conceptualization, Methodology, Investigation, Writing – review & editing, Visualization. **Niels P. Riksen:** Conceptualization, Methodology, Resources, Writing – review & editing, Supervision, Funding acquisition.

## Conflict of interest

MGN declares that he is one of the scientific founders of Trained Therapeutics Discovery (TTxD), Biotrip, Salvina and Lemba therapeutics. All other authors declare no competing interests.

## Data Availability

The dataset used and/or analyzed during the current study is available from the corresponding author upon reasonable request.
