# Supplementary material for: Innate immune cell function in statin-treated patients with severe hypercholesterolemia is comparable to normocholesterolemic individuals: A cross-sectional study
Source: Atheroscler Plus. 2025 Dec 4;62:53–61. doi: 10.1016/j.athplu.2025.11.004 (PMC12750502; doi:10.1016/j.athplu.2025.11.004)
Supplement: Multimedia component 1 [file mmc1.docx]

**Supplementary data**

**Supplementary figure 1: Timeline of inclusion date of patient timepoints.**
In gray the two major lockdowns in the Netherlands during the COVID19 pandemic

**Supplementary figure 2. Panel A: Gating strategy defining monocyte population using flow cytometry.**
Manual gating strategy for monocyte subsets: single cells (A) and CD45+ immune cells(B). Gating for monocytes, lymphocytes and granulocytes based on forward and side scatter(C). We excluded B-cells based on high expression of CD19(D). Thereafter, monocytes were identified based on the expression of CD14 and CD16 (E-F) HLA-DR+ monocytes were selected to eliminate NK cells (G). Monocyte subsets (classical, intermediate and nonclassical) were determined based on CD14 and CD16 expression and the exact gates were put based on HLA-DR and CCR2 expression (highest on nonclassical monocytes) (H-J).

**Panel B: Gating strategy for defining neutrophil population using flow cytometry.**
Manual gating strategy for neutrophil sub-analysis: gating for (A) single cells and (B) CD45+. Then granulocytes were selected based FSC/SSC (C). Eosinophils and basophils were eliminated based on low CD16 expression, and high CD123 expression respectively (D-E). Mature neutrophils were determined based on CD10 expression. (F) Neutrophils were further analyzed for their median fluorescent intensity of activation markers CD62L (G) and HLA-DR (H).

**Supplementary table 1. Flow cytometry antibodies**

| **Flow cytometry markers** |  |  |  |  |
| --- | --- | --- | --- | --- |
| **Antibodies** | **Fluorochrome** | **Clone** | **Company** | **Identifyer** |
| Anti-human CD16 | FITC | 3G8 | Biolegend | Cat# 302006  RRID AB_314206 |
| Anti-human HLA-DR | PE | immu-357 | Beckman Coulter | Cat# IM1639U RRID AB_2876782 |
| Anti-human CD11c | PEDazzle584 | BU15 | Biolegend | Cat# 337227 RRID AB_2564548 |
| Anti-human CD14 | PC7 | 61D3 | eBioscience | Cat# 25-0149 RRID AB_1582276 |
| Anti-human CD56 | APC | N901 | Beckman Coulter | Cat# IM2474 RRID AB_130791 |
| Anti-human CD19 | AF700 | HIB19 | Biolegend | Cat# 302226 RRID AB_493751 |
| Anti-human CD3 | PC5.5 | UCHT1 | Biolegend | Cat# 300410 RRID AB_314064 |
| Anti-human CCR2 | BV421 | 48607 | BD Biosciences | Cat# 564067 RRID AB_2738573 |
| Anti-human CD45 | BV510 | HI30 | Biolegend | Cat# 304036 RRID AB_2561940 |
| Anti-human CD11b | BV785 | ICRF44 | Biolegend | Cat# 301346 RRID AB_2563794 |
| Anti-human CD62L | PEDazzle584 | DREG-56 | Biolegend | Cat# 304842 RRID AB_2565874 |
| Anti-human CD49d | PECy5.5 | 9F10 | Biolegend | Cat# 304312 RRID AB_10641699 |
| Anti-human CD10 | PC7 | HI10a | Biolegend | Cat# 312213 RRID AB_2146549 |
| Anti-human lineage cocktail | APC | UCHT1; HCD14; HIB19; 2H7; HCD56 | Biolegend | Cat# 348703 RRID N/A |
| (CD3, CD14, CD19, CD20, CD56) |  |  |  |  |
| Anti-human CD66b | APC-700 | G10F5 | Biolegend | Cat# 305114 RRID AB_2566038 |
| Anti-human CD15 | APC-Cy7 | MEM-166 | Biolegend | Cat# 323047 RRID AB_2750189 |
| Anti-human CD123 | BV421 | 6H6 | Biolegend | Cat# 306018 RRID AB_10962571 |
| Anti-human CD35 | BV650 | E11 | BD Bioscience | Cat# 744277 RRID AB_2742115 |
| Brilliant stain buffer | - | - | BD Bioscience | Cat# 563794 RRID N/A |
| Helix NP^TM^ NIR | - | - | Biolegend | Cat# 425301 RRID N/A |

**Supplementary table 2. ELISA assays**

| **ELISA assays** | **Product number** | **Manufacturer** |
| --- | --- | --- |
| Human IL-1b DuoSet ELISA | DY201 | Bio-Techne/R&D |
| Human IL-1RA DuoSet ELISA | DY280 | Bio-Techne/R&D |
| Human IL-6 DuoSet ELISA | DY206 | Bio-Techne/R&D |
| Human IL-10 DuoSet ELISA | DY217B | Bio-Techne/R&D |
| Human TNF DuoSet ELISA | DY210 | Bio-Techne/R&D |
| Human hsCRP ELISA | DY1707 | Bio-Techne/R&D |
| Human IL-8 DuoSet ELISA | DY208 | Bio-Techne/R&D |
| Human Neutrophil Elastase/ELA2 DuoSet ELISA | DY9167 | Bio-Techne/R&D |
| Human S100A8/S100A9 Heterodimer DuoSet ELISA | DY8226 | Bio-Techne/R&D |
| Human Myeloperoxidase DuoSet ELISA | DY3174 | Bio-Techne/R&D |
| Human Lipocalin-2/NGAL DuoSet ELISA | DY1757 | Bio-Techne/R&D |

**Supplementary table 3. Primers for ChIP-qPCR**

| **TNFA promotor region** | 1. Forward: CAGGCAGGTTCTCTTCCTCT 1. Reverse: GCTTTCAGTGCTCATGGTGT | 2. Forward: AGAGGACCAGCTAAGAGGGA 2. Reverse: AGCTTGTCAGGGGATGTGG | 3. Forward: GTGCTTGTTCCTCAGCCTCT 3. Reverse: ATCACTCCAAAGTGCAGCAG |
| --- | --- | --- | --- |
|  | 4. Forward: TGTCTGGCACACAGAAGACA 4. Reverse: CCCTGAGGTGTCTGGTTTTC | 5. Forward: AGCCAGCTGTTCCTCCTTTA 5. Reverse: TTAGAGAGAGGTCCCTGGGG | 6. Forward: TGATGGTAGGCAGAACTTGG 6. Reverse: ACTAAGGCCTGTGCTGTTCC |
| **Myoglobulin negative control for H3K4me3** | 1. Forward: AGCATGGTGCCACTGTGCT 1. Reverse: GGCTTAATCTCTGCCTCATGAT |  |  |
| **GAPDH positive control for H3K4me3** | 1. Forward: CCCGGTTTCTATAAATTGAGC 1. Reverse: AAGAAGATGCGGCTGACTGT |  |  |

**Supplementary Table 4: Prescribed lipid lowering treatment**

| **Initial prescribed statin** | **No. of patients (n=21)** |
| --- | --- |
| Rosuvastatin | 17 |
| Atorvastatin | 2 |
| Rosuvastatin + Ezetimibe | 1 |
| Atorvastatin + Ezetimibe | 1 |
| **Initial prescribed statin dosage** | **No. of patients (n=21)** |
| Rosuvastatin 5 mg/day | 1 |
| Rosuvastatin 10 mg/day | 11 |
| Rosuvastatin 20 mg/day | 3 |
| Rosuvastatin 40 mg/day | 1 |
| Rosuvastatin 20 mg/day + Ezetimibe 10 mg/day | 2 |
| Atorvastatin 10 mg/day | 1 |
| Atorvastatin 40 mg/day | 1 |
| Atorvastatin 80 mg/day + Ezetimibe 10 mg/day | 1 |
|  |  |
| **Prescribed statin at 12 months** | **No. of patients (n=15)** |
| Rosuvastatin | 9 |
| Rosuvastatin + Ezetimibe | 1 |
| Atorvastatin + Ezetimibe | 4 |
| Fluvastatin + Ezetimibe | 1 |
| **Prescribed statin dosage at 12 months** | **No. of patients (n=15)** |
| Rosuvastatin 10 mg/day | 6 |
| Rosuvastatin 20 mg/day | 2 |
| Rosuvastatin 40 mg/day | 1 |
| Rosuvastatin 20 mg/day + Ezetimibe 10 mg/day | 1 |
| Atorvastatin 10 mg/day + Ezetimibe 10 mg/day | 1 |
| Atorvastatin 40 mg/day + Ezetimibe 10 mg/day | 2 |
| Atorvastatin 80 mg/day + Ezetimibe 10 mg/day | 1 |
| Fluvastatin 40 mg/day + Ezetimibe 10 mg/day | 1 |
|  |  |
| **Not completing study** | **No. of patients** |
| Lost to follow-up | 3 |
| With drawn informed consent | 1 |
| Patient switched to PCSK9 inhibitor | 1 |
| Patient stopped statin treatment: myalgia | 1 |

**Supplementary table 5:** **Cytokine production capacity of PBMC after 24 hours of stimulation. Healthy controls compared to treated patients.**

|  |  | **Healthy controls (n=18)** | **Treated patients (n=15)** |  |
| --- | --- | --- | --- | --- |
|  |  | **Mean ± SD** | **Mean ± SD** | ***p*-value** |
| **TNF (pg/ml)** | RPMI | 39 ± 0 | 39 ± 0 |  |
|  | LPS | 522 ± 643 | 313 ± 313 | 0.29 |
|  | P3C | 1000 ± 1146 | 748 ± 979 | 0.38 |
|  | MSU | 40 ± 2 | 39 ± 0 | >0.99 |
|  | Poly I:C | 79 ± 61 | 58 ± 23 | 0.36 |
|  | Candida Albicans | 4468 ± 1739 | 2998 ± 1304 | 0.02 |
| **IL1b (pg/ml)** | RPMI | 42 ± 9 | 39 ± 0 | 0.49 |
|  | LPS | 5027 ± 2798 | 4289 ± 2510 | 0.32 |
|  | P3C | 6605 ± 3067 | 5162 ± 2833 | 0.20 |
|  | MSU | 48 ± 39 | 39 ± 0 | 0.72 |
|  | LPS + MSU | 8607 ± 4967 | 6878 ± 3664 | 0.32 |
|  | Poly I:C | 143 ± 122 | 84 ± 51 | 0.24 |
|  | Candida Albicans | 7787 ± 2286 | 6631 ± 1819 | 0.17 |
| **IL6 (pg/ml)** | RPMI | 47 ± 0 | 47 ± 0 |  |
|  | LPS | 433 ± 440 | 301 ± 359 | 0.40 |
|  | P3C | 8832 ± 4316 | 8259 ± 4083 | 0.73 |
|  | MSU | 66 ± 80 | 47 ± 0 | >0.99 |
|  | Poly I:C | 68 ± 52 | 48 ± 2 | 0.70 |
|  | Candida Albicans | 1800 ± 1107 | 294 ± 188 | <0.00 |
| **IL 1Ra (pg/ml)** | RPMI | 10002 ± 5824 | 11911 ± 7103 | 0.42 |
|  | LPS | 13223 ± 5344 | 12226 ± 5688 | 0.66 |
|  | P3C | 18512 ± 5596 | 16714 ± 8023 | 0.24 |
|  | MSU | 11147 ± 6906 | 9709 ± 6728 | 0.58 |
|  | Poly I:C | 12304 ± 4392 | 9930 ± 5138 | 0.09 |
|  | Candida Albicans | 23739 ± 6131 | 17285 ± 7077 | 0.004 |
| **IL10 (pg/ml)** | RPMI | 35 ± 0 | 35 ± 0 |  |
|  | LPS | 95 ± 78 | 99 ± 93 | 0.85 |
|  | P3C | 164 ± 123 | 126 ± 49 | 0.90 |
|  | MSU | 35 ± 0 | 35 ± 0 | >0.99 |
|  | Poly I:C | 35 ± 0 | 35 ± 0 | >0.99 |
|  | Candida Albicans | 64 ± 35 | 46 ± 18 | 0.09 |

Data are presented as mean ± SD. *p*-value <0.05 was considered significant, using the Mann-Whitney statistical test. Abbreviations: TNF (tumor necrosis factor); IL-1b (interleukin-1 beta); IL1-Ra (interleukin-1 receptor antagonist); IL-6 (interleukin-6); IL-10 (interleukin-10); RPMI (Roswell Park Memorial Institute culture medium); LPS (lipopolysaccharide E. Coli); P3C (Pam3Cys); MSU (Monosodium urate crystals); Poly I:C (Polyinosinic:polycytidylic acid)

**Supplementary table 6: Calculated percentage of input (H3K4me3) for ChIP-qPCR.**

| **% of input (recovery)** | | | | | | | | |
| --- | --- | --- | --- | --- | --- | --- | --- | --- |
| **Group** | **Negative locus control** | **Positive locus control** | **Primer 1** | **Primer 2** | **Primer 3** | **Primer 4** | **Primer 5** | **Primer 6** |
| **HC** | 0.025974 | 8.388919 | 6.657732 | 5.24711 | 7.80526 | 3.91802 | 2.880134 | 1.578132 |
| **HC** | 0.011085 | 5.617829 | 5.72863 | 3.914003 | 5.011169 | 2.752093 | 2.378797 | 1.040041 |
| **HC** | 0.02339 | 5.433784 | 3.042953 | 2.328662 | 4.025511 | 1.395761 | 1.026288 | 0.582514 |
| **HC** | 0.004298 | 7.004076 | 4.90042 | 3.868295 | 7.063955 | 2.662551 | 1.965532 | 1.023879 |
| **HC** | 0.029705 | 9.942402 | 6.744651 | 5.679401 | 9.863469 | 4.007759 | 3.488918 | 1.944367 |
| **HC** | 0.033399 | 10.74722 | 7.951798 | 5.934701 | 10.0218 | 3.909157 | 3.330411 | 2.350514 |
| **HC** | 0.021371 | 13.34474 | 7.710022 | 5.723194 | 8.8009 | 3.372687 | 2.406609 | 1.683034 |
| **HC** | 0.117346 | 12.94783 | 9.938985 | 7.633951 | 11.62931 | 5.298227 | 4.214638 | 2.301784 |
| **HC** | 0.066878 | 9.082166 | 8.631203 | 4.864929 | 9.975991 | 5.357394 | 3.153211 | 2.323529 |
| **HC** | 0.071508 | 10.17873 | 7.557681 | 5.213952 | 7.307417 | 4.196656 | 3.323875 | 2.244442 |
| **HC** | 0.018084 | 7.49218 | 3.415765 | 0.485339 | 4.835861 | 2.089047 | 2.170068 | 1.136671 |
| **HC** | 0.028853 | 10.67532 | 5.975555 | 5.245133 | 6.130937 | 2.692644 | 2.194537 | 1.109823 |
| **HC** | 0.027404 | 7.047379 | 4.504162 | 4.683715 | 6.127169 | 2.695247 | 2.256683 | 1.228958 |
| **HC** | 0.03724 | 2.546821 | 1.790384 | 2.18203 | 2.905929 | 1.070566 | 0.770818 | 0.366561 |
| **HC** | 0.018646 | 11.30207 | 18.3899 | 4.826217 | 16.09143 | 5.081709 | 7.3319 | 3.007259 |
| **HC** | 0.014457 | 3.532538 | 4.080158 | 1.661285 | 4.285462 | 1.620438 | 1.364162 | 0.46315 |
| **HC** | 0.034352 | 2.299026 | 3.052309 | 0.901832 | 3.111662 | 1.293796 | 1.030001 | 0.949139 |
| **PT** | 0.038471 | 7.851326 | 4.470299 | 3.524053 | 0.978797 | 1.364256 | 1.729795 | 1.219964 |
| **PT** | 0.032572 | 5.116291 | 8.345214 | 5.893519 | 7.609546 | 1.267352 | 1.657704 | 1.644928 |
| **PT** | 0.038471 | 2.905272 | 2.736133 | 1.60483 | 3.299749 | 1.026957 | 1.155228 | 0.527213 |
| **PT** | 0.011587 | 3.922711 | 2.559786 | 2.205983 | 4.425772 | 1.204853 | 1.081637 | 0.637172 |
| **PT** | 0.038471 | 2.621513 | 1.368434 | 1.055105 | 1.715926 | 0.592015 | 0.670516 | 0.319786 |
| **PT** | 0.038471 | 7.307755 | 6.555099 | 1.798704 | 6.95289 | 2.839967 | 2.126616 | 1.337162 |
| **PT** | 0.016166 | 13.40232 | 12.81749 | 11.01454 | 18.60638 | 6.604291 | 5.271111 | 3.231139 |
| **PT** | 0.012073 | 9.865738 | 7.838538 | 6.495985 | 10.34692 | 3.821186 | 3.615579 | 2.212245 |
| **PT** | 0.021215 | 11.71655 | 9.266082 | 7.172412 | 11.14152 | 4.25258 | 4.484364 | 3.101833 |
| **PT** | 0.008437 | 6.861146 | 6.344649 | 3.880594 | 6.347695 | 2.848307 | 3.828833 | 1.33223 |
| **PT** | 0.03518 | 11.97018 | 9.352765 | 6.123404 | 11.51586 | 5.070951 | 3.693607 | 2.780428 |
| **PT** | 0.08394 | 23.48787 | 16.74562 | 10.63305 | 14.41465 | 8.558638 | 6.303589 | 3.762425 |
| **PT** | 0.03705 | 18.9185 | 12.5701 | 7.505525 | 15.27998 | 8.279628 | 7.056012 | 3.212487 |
| **PT** | 0.105352 | 12.04067 | 9.146742 | 6.421849 | 12.79968 | 3.832023 | 3.466729 | 2.553364 |
| **PT** | 0.059607 | 10.13406 | 8.338652 | 6.283386 | 8.48729 | 3.55218 | 2.960157 | 1.819664 |

*Abbreviations: HC healthy control, PT patients*

**Supplementary table 7:** **Cytokine production capacity of neutrophils after 4 hours stimulation. Healthy controls compared to treated patients.**

|  |  | **Healthy controls (n=18)** | **Treated patients (n=14)** |  |
| --- | --- | --- | --- | --- |
|  |  | **Mean ± SD** | **Mean ± SD** | ***p*-value** |
| **MPO** | **RPMI** | 106257 ± 46637 | 107913 ± 41010 | 0.99 |
|  | **LPS** | 176337 ± 115831 | 119887 ± 59503 | 0.16 |
|  | **P3C** | 100178 ± 42446 | 97361 ± 26179 | 0.99 |
|  | **MSU** | 140353 ± 71552 | 111842 ± 46516 | 0.34 |
|  | **LPS+MSU** | 155802 ± 60254 | 116915 ± 35748 | 0.06 |
|  | **Nigericin** | 303550 ± 112534 | 217079 ± 58432 | 0.01 |
|  | **PMA** | 354653 ± 99248 | 309591 ± 73671 | 0.40 |
| **S100 A8/9** | **RPMI** | 1536175 ± 1198995 | 1200783 ± 305128 | 0.87 |
|  | **LPS** | 1696918 ± 683613 | 1116985 ± 323482 | 0.01 |
|  | **P3C** | 1420617 ± 452414 | 1268285 ± 312165 | 0.38 |
|  | **MSU** | 2338788 ± 823237 | 1839336 ± 729771 | 0.12 |
|  | **LPS+MSU** | 2668065 ± 1231616 | 1778597 ± 563339 | 0.04 |
|  | **Nigericin** | 3592223 ± 1367451 | 2813502 ± 1522196 | 0.05 |
|  | **PMA** | 6446147 ± 2094704 | 6503364 ± 3943558 | 0.28 |
| **NGAL** | **RPMI** | 23640 ± 10069 | 25897 ± 10237 | 0.49 |
|  | **LPS** | 37296 ± 15102 | 31280 ± 14479 | 0.27 |
|  | **P3C** | 71206 ± 25375 | 65193 ± 17654 | 0.69 |
|  | **MSU** | 41705 ± 15731 | 26979 ± 13649 | 0.01 |
|  | **LPS+MSU** | 44987 ± 15938 | 30718 ± 12159 | 0.02 |
|  | **Nigericin** | 55273 ± 24774 | 41173 ± 19936 | 0.05 |
|  | **Ethanol control for nigericin** | 29890 ± 11389 | 22643 ± 10164 | 0.05 |
|  | **PMA** | 237102 ± 27591 | 217217 ± 22750 | 0.08 |
| **IL-8** | **RPMI** | 47 ± 0 | 47 ± 0 | >0.99 |
|  | **LPS** | 48.6 ± 4 | 48.8 ± 5 | 0.85 |
|  | **PMA** | 158.3 ± 110 | 143.6 ± 110 | 0.44 |

Data are presented as mean ± SD. *p*-value <0.05 was considered significant, using the Mann-Whitney statistical test.
Abbreviations: MPO (Myeloperoxidase); S100A8/9 (S100 calcium-binding protein A8/9 heterodimer); NGAL (Neutrophil Gelatinase Associated Lipocalin); RPMI (Roswell Park Memorial Institute culture medium); LPS (lipopolysaccharide E. Coli); P3C (Pam3Cys); MSU (Monosodium urate crystals); Nigericin (an NLRP3 inflammasome inducer); PMA (Phorbol-12-myristate-13-acetate).
