# Supplementary figures and images for: Innate immune cell function in statin-treated patients with severe hypercholesterolemia is comparable to normocholesterolemic individuals: A cross-sectional study
Source: Atheroscler Plus. 2025 Dec 4;62:53–61. doi: 10.1016/j.athplu.2025.11.004 (PMC12750502; doi:10.1016/j.athplu.2025.11.004)

## Slide 1
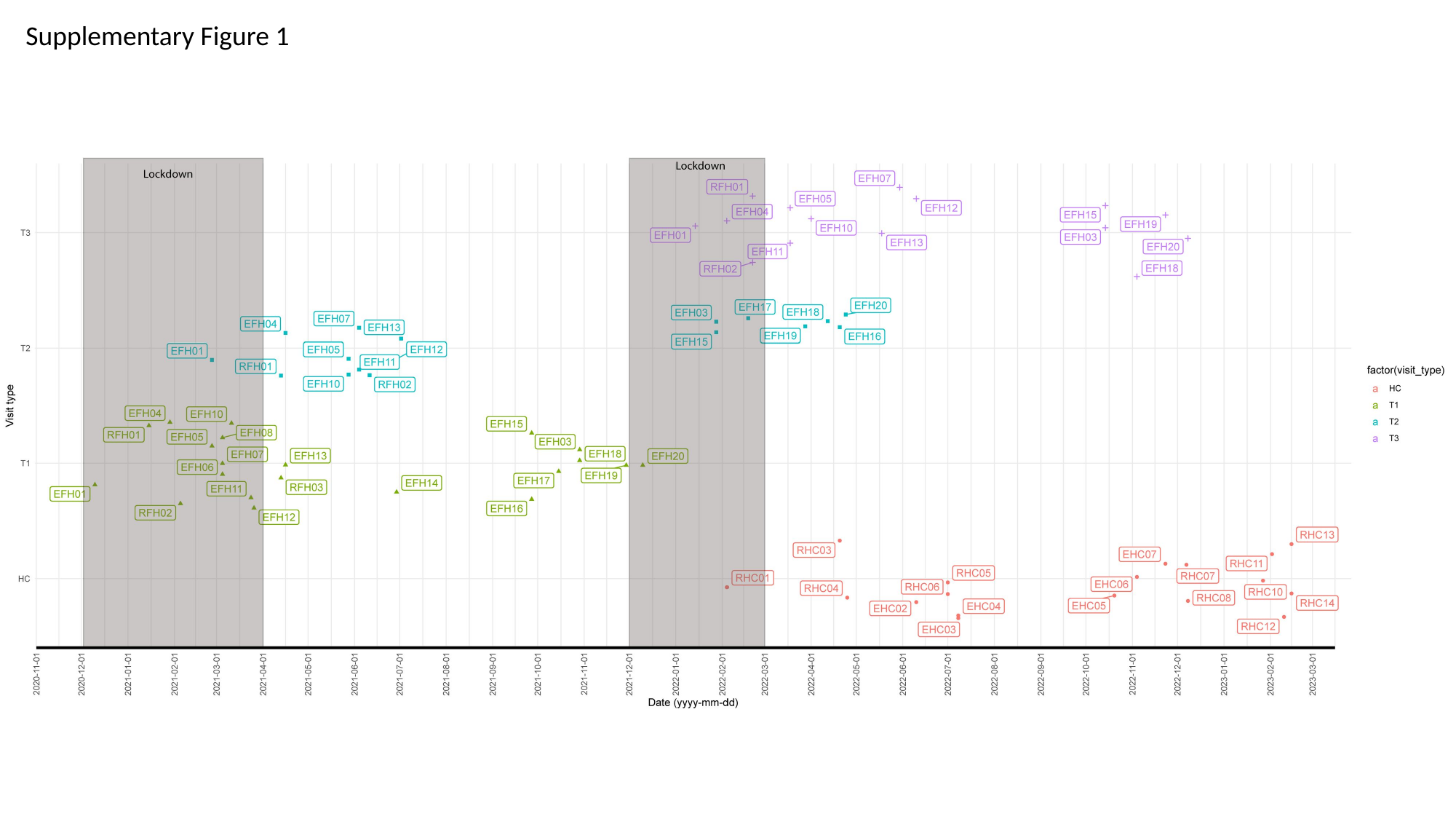

Supplementary Figure 1

## Slide 2
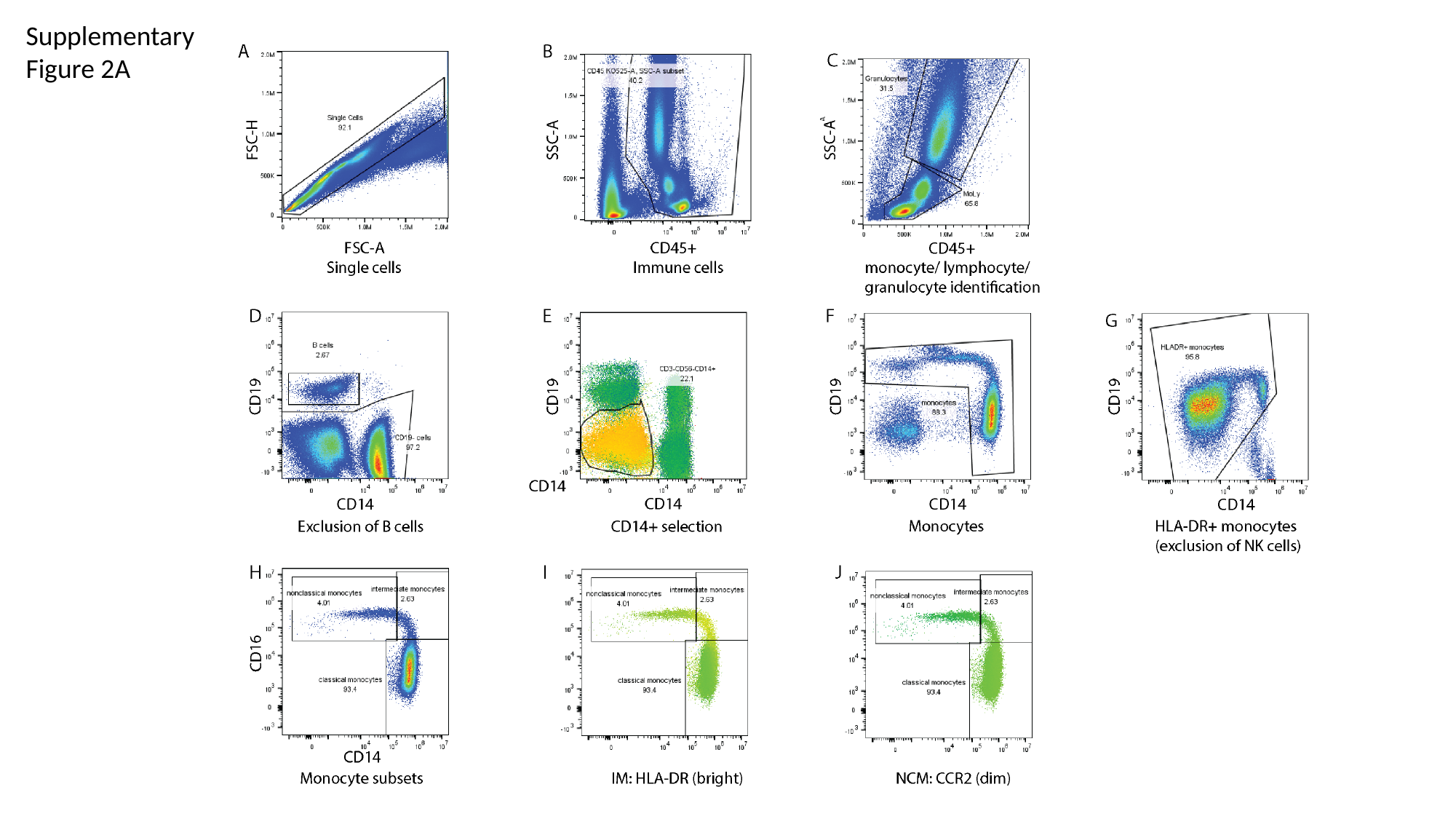

Supplementary Figure 2A

## Slide 3
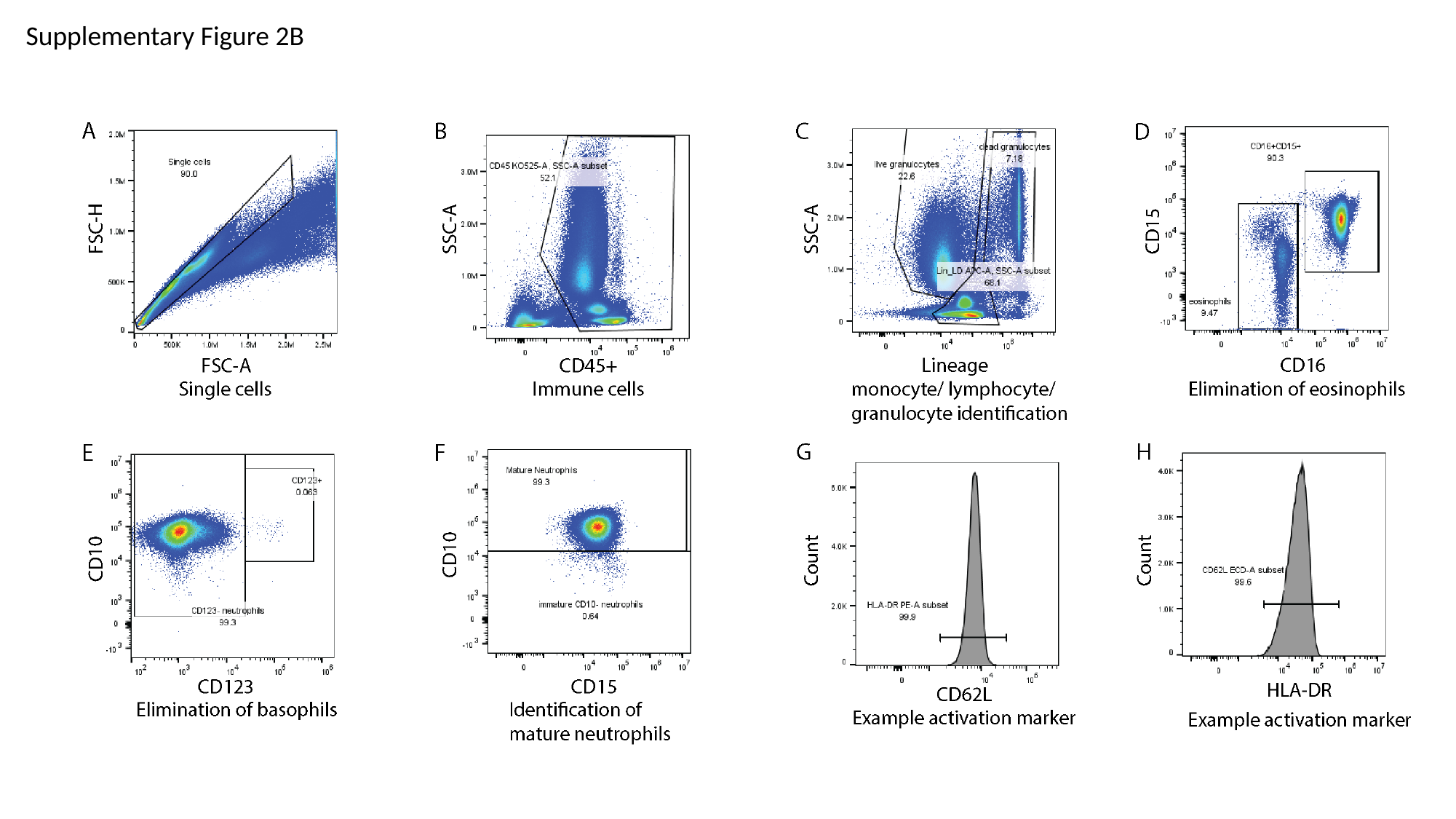

Supplementary Figure 2B

Supplement: Multimedia component 2 [file mmc2.pptx]
